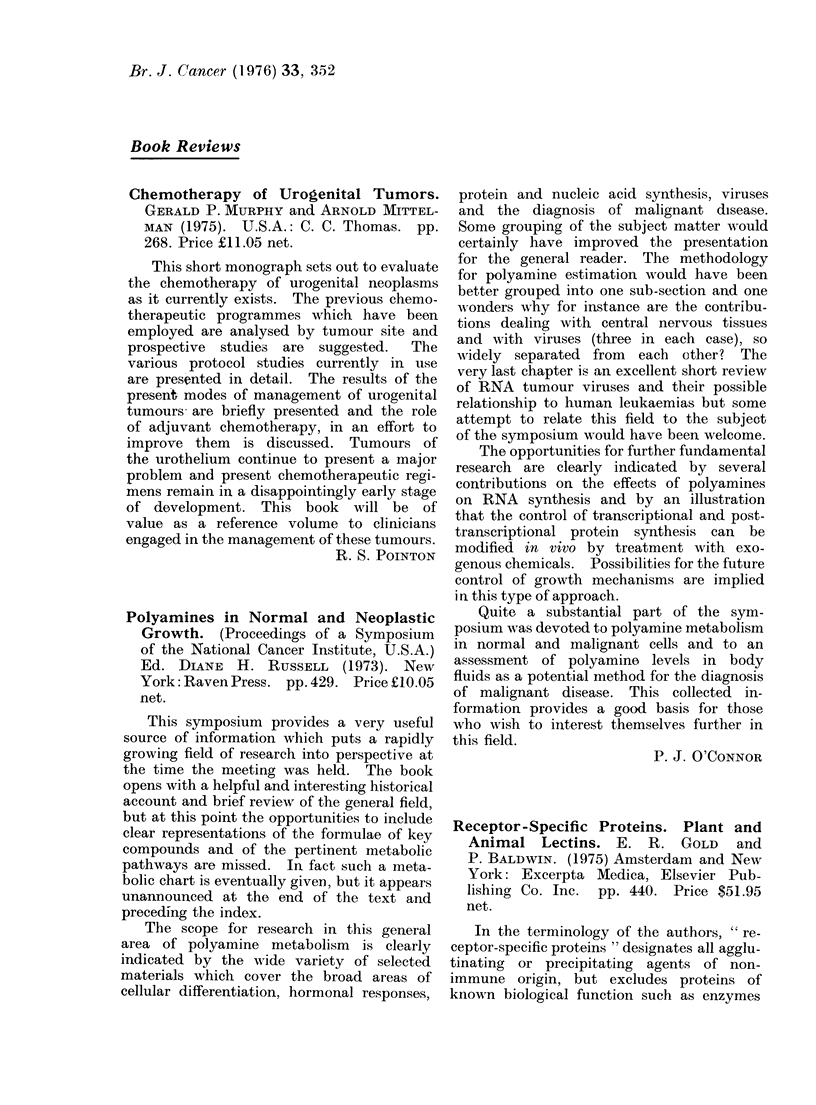# Polyamines in Normal and Neoplastic Growth

**Published:** 1976-03

**Authors:** P. J. O'Connor


					
Polyamines in Normal and Neoplastic

Growth. (Proceedings of a Symposium
of the National Cancer Institute, U.S.A.)
Ed. DIANE H. RUSSELL (1973). New
York: Raven Press. pp. 429. Price ?10.05
net.

This symposium provides a very useful
source of information which puts a rapidly
growing field of research into perspective at
the time the meeting was held. The book
opens with a helpful and interesting historical
account and brief review of the general field,
but at this point the opportunities to include
clear representations of the formulae of key
compounds and of the pertinent metabolic
pathways are missed. In fact such a meta-
bolic chart is eventually given, but it appears
unannounced at the end of the text and
preceding the index.

The scope for research in this general
area of polyamine metabolism is clearly
indicated by the wide variety of selected
materials which cover the broad areas of
cellular differentiation, hormonal responses,